# Smartphone Ocular Fundal Photography in the Diagnosis of Raised Intracranial Pressure: A Novel Adaptation to Neurosurgical Practice

**DOI:** 10.7759/cureus.38246

**Published:** 2023-04-28

**Authors:** Tochukwu H Mbanugo, Wilfred C Mezue, Jude-Kennedy C Emejulu, Enoch O Uche, Mark O Chikani, Izuchukwu Iloabachie, Ephraim Onyia, Udoka Okpalauwaekwe

**Affiliations:** 1 Surgery/Neurological Surgery, Nnamdi Azikiwe University Teaching Hospital, Nnamdi Azikiwe University, Nnewi, NGA; 2 Surgery/Neurological Surgery, University of Nigeria Teaching Hospital, University of Nigeria, Enugu, NGA; 3 Academic Family Medicine, University of Saskatchewan, Saskatoon, CAN

**Keywords:** resource-poor settings, papilledema, spontaneous retinal venous pulsation, intracranial pressure, smartphone ophthalmoscopy

## Abstract

Background

Consistently raised intracranial pressure (ICP) is a common final pathway to morbidity/mortality in many neurosurgical conditions. This underscores the need for early diagnosis and prompt management of raised ICP. This study aims to determine whether smartphone fundal photography features of raised ICP can accurately predict the computed tomography (CT) findings suggestive of elevated ICP in neurosurgery patients.

Methods

Dilated ocular fundal photography examinations using an ophthalmoscope adapter mounted on a smartphone were done on 82 patients with clinical suspicion of raised ICP. Fundal photography findings were recorded as pictures/videos for disc analysis. Patients subsequently had neuroimaging with results analyzed for radiological features of raised ICP. These were correlated with fundal photography findings.

Results

A total of 82 adult patients participated in this study. Chi-square analysis showed a relationship between radiological signs of raised ICP and the absence of spontaneous retinal venous pulsation (SRVP) (p=0.001). There was no relationship observed between papilledema and radiological signs of raised ICP. However, when the fundal photography signs were aggregated, there was a significant relationship between the fundal signs of raised ICP and radiological signs of raised ICP (p=0.004). The sensitivity and specificity of smartphone-fundoscopy-detected papilledema in predicting radiological signs of raised ICP were 43.2% and 100%, respectively, while those of absent SRVP were 100% and 92.6%, respectively.

Conclusion

Smartphone ophthalmoscopy is a reliable screening tool for evaluating ICP in neurosurgical patients. It should be introduced into the neurosurgeon's tools for prompt evaluation of raised ICP, especially in developing/resource-poor settings where CT or magnetic resonance imaging is not readily available.

## Introduction

Non-invasive modalities for diagnosing raised intracranial pressure (ICP) have been the subject of many research studies. Advantages of non-invasive modalities include avoidance of surgery and its attendant complications, lack of need for expensive, bulky equipment, and demand for skilled expertise required for invasive monitoring [1,2]. These conditions make invasive measures unattractive in resource-poor environments. Most patients with raised ICP in our environment are diagnosed or confirmed following radiological investigations such as cranial computed tomography (CT) scan or magnetic resonance imaging (MRI) scan. These radiological investigations are also not readily available or functional in many tertiary health institutions. Thus, it mostly requires patients' transportation to private neuroimaging outfits for the usually expensive investigations. The costs of these investigations impose a burden on these patients who have to meet the cost out of personal pocket. These factors contribute to delays in diagnosing and treating patients with raised ICP and underscore the need for simpler, more readily accessible, and less expensive non-invasive screening modalities. These can aid in prompt diagnosis of raised ICP and early treatment measures pending definitive diagnosis by radiological imaging.

Smartphone fundal photography uses a novel portable ophthalmoscope adapter to view and capture retinal images and videos through a dilated pupil with the help of a smartphone [3-5]. With this device, ocular signs of raised ICP such as the absence of spontaneous central retinal venous pulsation, papilledema, and retinal hemorrhages can be observed and used to screen for raised ICP [6-9].

Smartphone fundal photography presents an objective option for fundoscopy as images/videos can be stored, reviewed, and sent to other specialists for a second opinion. The images obtained from the smartphone fundal photography technique provide records and also serve as documentation for medicolegal purposes, telemedicine, teaching, and research [3-5,10,11]. It is a novel technique that prevents both the shortcomings of the subjective, conventional ophthalmoscope and the bulky, expensive traditional fundal camera in many ophthalmology departments. Smartphone photography is a new convenient hybrid technique that ensures portability and ease of use, providing the valuable option of storing the fundus/retina's images. There is a paucity of studies on the use of smartphone fundal cameras as a non-invasive assessment tool for assessing raised ICP among neurosurgical patients, especially in our environment.

Study objectives

The study aims to determine whether smartphone fundal photography features can accurately predict the computed tomographic (CT) findings suggestive of elevated ICP in neurosurgery patients. Cranial CT is chosen for this study because it is the most available modality and the only method of identifying raised ICP acutely in the emergency room. Our specific objectives were to determine the specificity, sensitivity, positive predictive values (PPVs), and negative predictive values (NPVs) of absent spontaneous retinal venous pulsation (SRVP) assessed using smartphone fundal photography in predicting features suggestive of raised ICP on cranial CT scan. We also pursued to determine the specificity, sensitivity, PPVs, and NPVs of present papilledema assessed using smartphone fundal photography in predicting features suggestive of raised ICP on cranial CT scan.

## Materials and methods

Study design and ethical considerations

We employed a prospective study set at the University of Nigeria Teaching Hospital, Enugu, Nigeria (UNTH), between June 2018 and July 2019. Ethical approval was sought and obtained from the Health Research Ethical Committee of the University of Nigeria Teaching Hospital, Enugu, before the commencement of the study, with approval IRB number, UNTH/CSA/329/OL.5. Written informed consent was obtained from all the subjects and relatives.

Setting, participants, and eligibility criteria

The University of Nigeria Teaching Hospital, Enugu, Nigeria (UNTH) is located in Ituku-Ozalla, a village, near Enugu city in Enugu state, Nigeria. Healthcare provision is mainly out of pocket (although private health insurance schemes exist) and, because of the distance from major cities in the state, is typically accessed by patients for tertiary use (specialist care). For this study, our inclusion criteria were adults aged 18 years and over, admitted as neurosurgical patients with clinical features of raised ICP (which is obtained from history and physical examination), with no contraindication for a CT scan. Exclusion criteria included prior use of steroids or mannitol to lower ICP, significant periorbital edema that will impede proper ocular fundal examination, prior orbital surgeries from primary orbital lesions, significant blunt orbital trauma, cataracts, and prior history of orbital radiation.

Data collection instruments and study procedures

Patient demographics (age and gender) were collected using a proforma, and presenting neurological status was determined using the Glasgow coma scale. Patients that presented with symptoms and signs of raised ICP from any cause were also included consecutively in the study.

Participants' pupils were dilated using one drop of tropicamide 1% and phenylephrine 2.5% per pupil when indicated. Typically, adequate pupillary dilatation was observed within 15-25 minutes of administration of topical mydriatic. After adequate pupillary dilation (≥5mm) was achieved, the patient was placed either supine or sitting, depending on the patient's clinical status and level of consciousness for the fundal examination.

**Figure 1:**
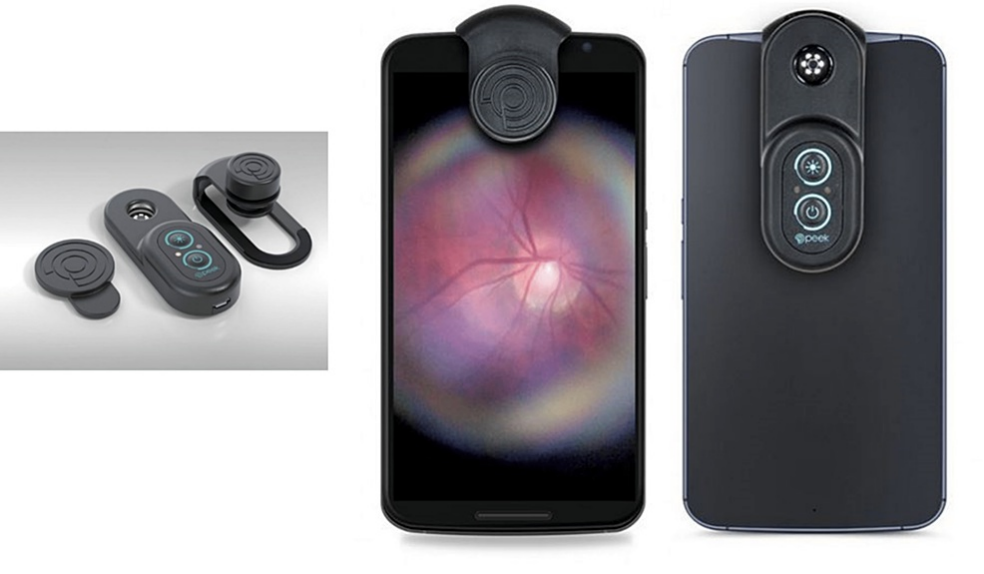
Peek Retina Ophthalmoscope adapter mounted on a smartphone Stock photos used with permission from Peek Vision Ltd (www.peekvision.org)

**Figure 2:**
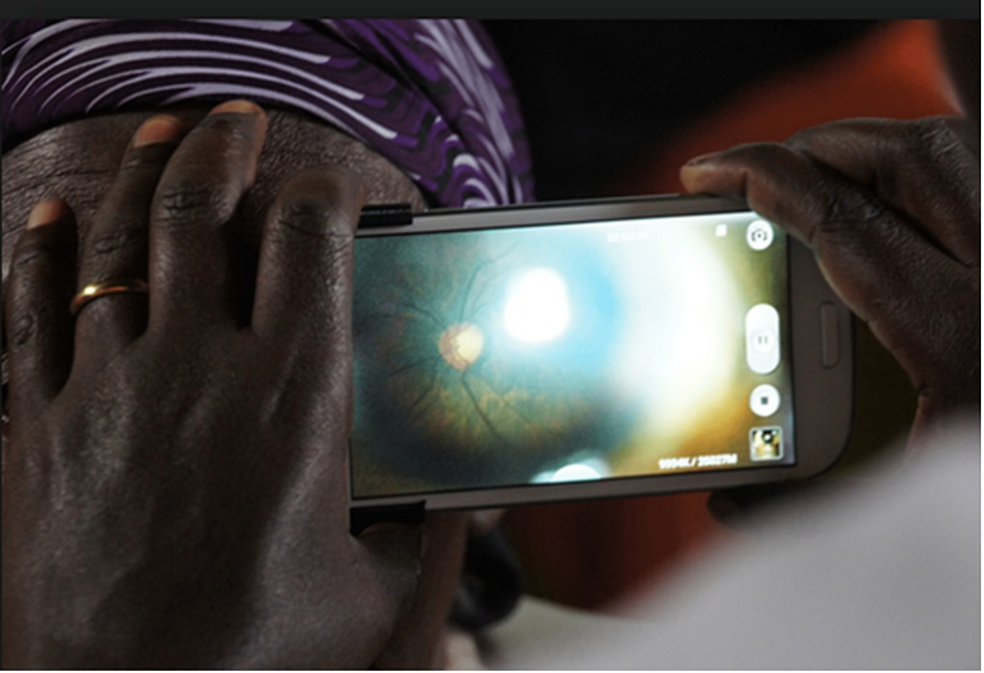
Smartphone ophthalmoscope in use

We used a smartphone-Tecno Camon X (TECNO CA7, Tecno, Shenzhen, China) running on Android "Oreo" version 8.1.0 software (with a native camera at 16 megapixels) with the Peek Retina ophthalmoscope adapter (www.peekvision.org; see Figure 1) to perform dilated pupillary fundal examinations on all the study participants. The use of the Peek retina ophthalmoscope smartphone adapter for clinical fundal examination has been validated in a multicenter study spearheaded by the London School of Hygiene and Tropical Medicine [11]. We recorded images as videos or pictures for disc analysis (see Figure 2). We also recorded the presence or absence of fundal signs of raised ICP (i.e., papilledema, absent spontaneous retinal venous pulsation, and retinal hemorrhages). Papilledema, when present, was graded using the Frisen grading system [12] (see Appendix 1). Stage one (very early papilledema) and above were considered relevant.

 

Each fundal examination lasted about one to three minutes, so it does not interfere significantly with the clinical management. Following admission and initial resuscitation, the patients were sent for neuroimaging, after which the results were collated in a proforma and analyzed. Radiological criteria considered as suggestive of raised ICP on cranial CT scan based on available literature include one or more mass effects with midline shift ≥3mm, hydrocephalus, collapsed third or fourth ventricle, effacement of sulci with evidence of cerebral edema, abnormal basal (mesencephalic) cisterns, and ipsilateral lateral ventricle compression with contralateral lateral ventricle dilatation. Fundal examinations were done before neuroimaging to mitigate observer bias. The lead researcher was blinded to all neuroimaging reports. The fundal photography findings and raised ICP radiological findings were analyzed using identified statistical methods.

Data analysis

Statistical analyses were done using IBM SPSS Statistics for Windows, Version 21 (Released 2012; IBM Corp., Armonk, New York, United States). Descriptive analyses were completed for clinical presentation signs and symptoms (i.e., fundal findings, raised ICP radiological profile, papilledema (presence or absence), SRVP (presence or absence), retinal hemorrhage (presence or absence), and fundal signs of raised ICP (presence or absence). Specificity, sensitivity, PPVs, and NPVs were calculated for each independent clinical test (i.e. papilledema, absent SRVP, and fundal signs of raised ICP) compared with CT radiological findings. Statistical significance was set at a level of significance of 5%.

## Results

Descriptive findings

Demographic Profile

**Figure 3:**
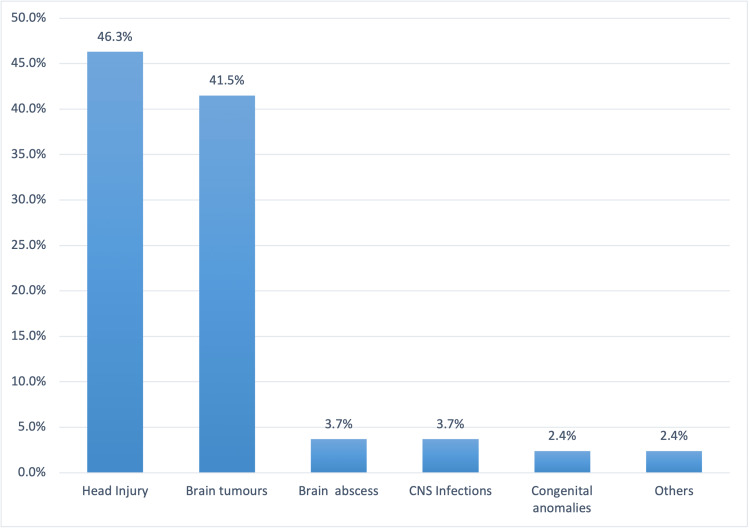
Distribution of fundal findings in the clinical groups CNS: Central nervous system

Eighty-two patients were recruited for this study, 60 males (73.2%) and 22 females (26.8%). The mean age of patients in this series was 38.7 years (±21.3). The cause of raised ICP in this series was broadly divided into traumatic and non-traumatic etiologies. Traumatic etiology accounted for 47.6% (39 patients) while non-traumatic causes accounted for 52.4% (43 patients). The definitive diagnoses in this series are shown in Figure 3. Thirty-eight patients (46.3%) were diagnosed with head injury, 34 (47.6%) with a brain tumor, 3 (3.7%) with brain abscess and CNS infections independently, and 2 (2.4%) with a congenital anomaly (see Figure 3).

Papilledema was present in 21.1% (i.e., 8 out of 38 patients) of patients with a head injury, 70.6% (24 out of 34 patients) of patients with brain tumors, and 33.3% (1 out of 3 patients) of patients with brain abscesses and central nervous system infections. One out of two (50%) patients with congenital anomalies presented with papilledema.

**Figure 4:**
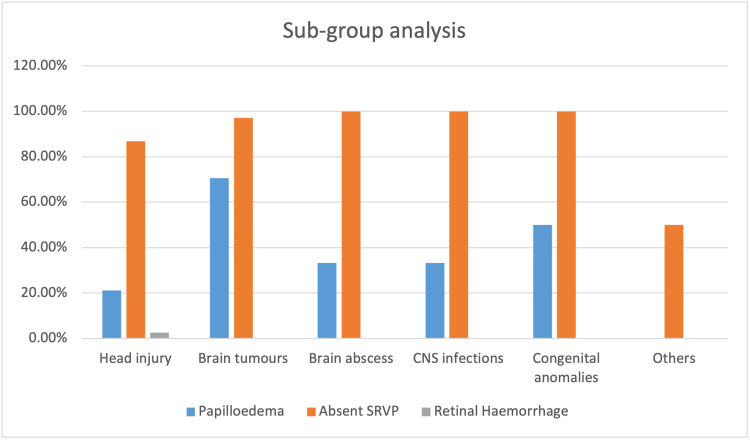
Incidence of different fundal findings in the diagnostic subgroups CNS: Central nervous system; SRVP: spontaneous retinal venous pulsation

SRVP was absent in 86.8% of head-injured patients, 97.1% of brain tumors, and all patients with brain abscesses and central nervous system infections. Retinal hemorrhage was seen in one head-injured patient in this series (see Figure 4).

Profile of radiological findings on Cranial CT scan

**Figure 5:**
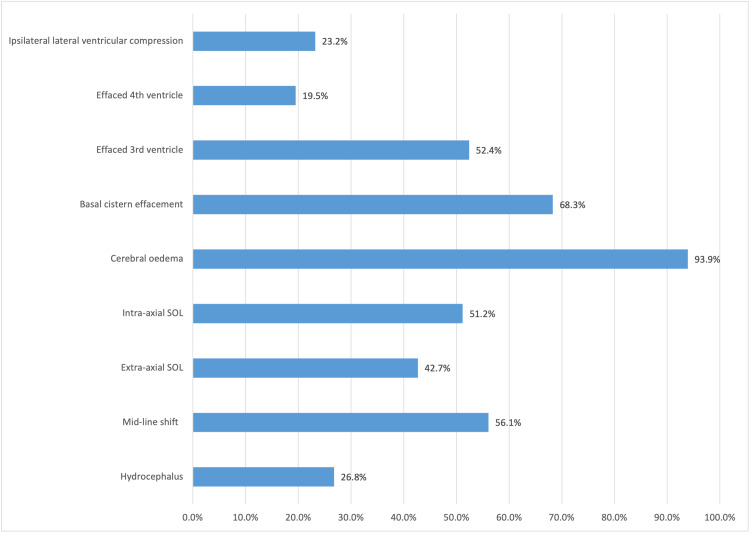
Profile of radiological signs of raised ICP on Cranial CT scan SOL: space-occupying lesion. Radiological signs of raised ICP: Hydrocephalus, mid-line shift >3mm, extra-axial space-occupying lesion, intra-axial space-occupying lesion, cerebral edema, basal cistern effacement, effaced 3rd ventricle, effaced 4th ventricle, and ipsilateral lateral ventricular compression with contralateral lateral ventricular dilatation.

All patients had radiological imaging with 81 patients (98.8%) having at least, one radiological sign of raised ICP. The chart below (see Figure 5) shows the representation of the radiological signs of raised ICP in this series.

Univariate analysis

Chi-square analysis of independence performed to evaluate the relationship between radiological signs of raised ICP and the absence of SRVP was statistically significant with a p-value of 0.001 (chi-square= 10.847; see Table 1). There was no significant relationship between papilledema or retinal hemorrhage and radiological signs of raised ICP. However, when the fundal signs were aggregated, there was a significant relationship between the fundal signs of raised ICP and radiological signs of raised ICP (p-value 0.004, chi-square =8.211, see Table 1).

**Table 1:** Relationship between radiological signs of raised ICP and fundal signs Intracranial pressure; SRVP: Spontaneous retinal venous pulsation. *p-value significant at 0.05

Variable (n=82)	Radiological signs (Present)	Radiological signs (Absent)	c^2^	p-value
Papilledema			0.754	0.385
Present	35	0		
Absent	46	1		
SRVP			10.847	0.001*
Present	0	1		
Absent	75	6		
Retinal hemorrhage			0.012	0.911
Present	1	0		
Absent	80	1		
Fundal signs of raised ICP			8.211	0.004*
Present	73	0		
Absent	8	8		

Predictive capacity of clinical tests

The sensitivity of smartphone fundoscopy-detected-papilloedema in predicting radiological signs of raised ICP was 43.2%, while the specificity was 100%. The absence of SRVP had a sensitivity of 100% and a specificity of 92.6% in predicting radiological signs of raised ICP. The presence of smartphone ophthalmoscopy features of raised ICP had an overall sensitivity of 90.1% and specificity of 100% in predicting the presence of cranial CT findings of raised ICP. The PPVs and the NPVs are also outlined in Table 2.

**Table 2:** Predictive value of papilloedema, SRVP, and fundal signs of ICP against the radiological confirmation of raised ICP (N=82) ICP: Intracranial pressure; NPV: negative predictive value; PPV: positive predictive value; SRVP: spontaneous retinal venous pulsation

Clinical tests/investigation	PPV (%)	NPV (%)	Sensitivity (%)	Specificity (%)
Papilledema	100	2.1	43.2	100
Absent SRVP	14.3	100	100	92.6
Fundal signs of raised ICP	100	11.1	90.1	100

## Discussion

Raised ICP is well known to play a critical role in the final pathway of both traumatic brain injuries and non-traumatic brain pathologies [2,13]. Elevated ICP is a frequent neurological emergency in patients with brain tumors and, when not promptly diagnosed and managed rapidly, results in irreversible neurologic deficits and death [14]. Fundal signs of raised ICP (papilledema and absent SRVP were found to have a positive correlation with radiological signs of raised ICP (p = 0.004). This indicates the smartphone ophthalmoscopy's ability to detect fundal features of raised ICP. Adam et al. (2015), in their study of the diagnostic utility of smartphone ophthalmoscopy, showed that it produces images approaching the quality and diagnostic utility of traditional fundal cameras [15]. Other studies reported similar findings [4,11,16].

Papilledema was very specific (100%) in predicting radiographic signs of raised ICP but poor in sensitivity (43%). While all patients with papilledema in this study had signs of raised ICP on cranial CT scans, the absence of papilledema did not rule out raised ICP in up to 57% of patients. Using papilledema alone as an indicator of raised ICP could lead to an unacceptably high rate of false-negative cases, especially among head-injured patients.

Absent SRVP was found to have a significant relationship with raised ICP findings on cranial CT scans (p-value 0.001). This relationship between absent SRVP and raised ICP has been well-reported in the literature [9,17-24]. Absent SRVP had a sensitivity and a specificity of 100% and 92.6%, respectively, in predicting cranial CT findings of raised ICP in this study. The presence of SRVP is highly sensitive for the patient not having raised ICP. Retinal venous pulsation is seen in 85-94% of people with normal ICP though this value rises to about 99% with the use of infrared retinal cameras [9,18,22-24].

The aggregate of fundal findings of raised ICP (papilledema, absent SRVP, retinal hemorrhages) on smartphone ophthalmoscopy has a sensitivity of 90.1% and a specificity of 100% in predicting the presence of signs of raised ICP on cranial CT scans. Our research findings underscore smartphone ophthalmoscopy's possible role and utility in the routine evaluation of neurosurgery patients with suspicion of raised ICP. One out of every two physicians uses smartphones [16]. This readily available piece of technology can be adapted to great advantage (especially in our resource-constrained environment) in screening neurosurgical patients for raised ICP. This will ensure prompt and objective neuro-ophthalmologic evaluation of patients for raised ICP and subsequent treatment, especially where cranial CT scans or MRI scans are not readily available. The value of this modality, especially in the acute setting, cannot be over-emphasized. The transient loss of ability to monitor pupillary size and the reaction was not found to affect management decisions because the fundoscopy results provide decision information.

Study limitations

This study is not without some limitations. We could not ascertain SRVP in all patient participants; for example, the pre-morbid state of SRVP in the patients could not be determined since there is an absence of SRVP in about 5-10% of the normal population. Secondly, we could not eliminate the observer bias that may have arisen in the analyses of the fundal and radiological images. Nonetheless, we tried to minimize this by blinding the fundal image analysis to the neuroimaging report and by involving consultants/senior residents of ophthalmology and radiology in the study of images.

## Conclusions

Smartphone ophthalmoscopy is a reliable screening tool for evaluating ICP in neurosurgical patients. As a result of this, we recommend that smartphone ophthalmoscopy be introduced into the neurosurgeon's tools for prompt evaluation of raised ICP, especially in developing/resource-poor countries where neuroimaging modalities like CT and MRI are not readily available.

## Appendices

APPENDIX 1

Papilledema Grading System (Frisen Scale) [12]

Stage 0 - Normal optic disc blurring of nasal, superior, and inferior poles in inverse proportion to disc diameter. Radial nerve fiber layer (NFL) without NFL tortuosity. Rare obscuration of a major blood vessel, usually on the upper pole.

Stage 1 -Very early papilledema obscuration of the nasal border of the disc.  No elevation of the disc borders. Disruption of the normal radial NFL arrangement with grayish opacity accentuating nerve fiber layer bundles. Normal temporal disc margin. Subtle grayish halo with a temporal gap. Concentric or radial retro-choroidal folds.

Stage 2 - Early papilledema obscuration of all borders. Elevation of the nasal border. Complete peripapillary halo.

Stage 3 - Moderate papilledema obscurations of all borders. Increased diameter of optic nerve head. Obscuration of one or more segments of major blood vessels leaving the disc. Peripapillary halo-irregular outer fringe with finger-like extensions.

Stage 4 - Marked papilledema elevation of the entire nerve head. Obscuration of all borders. Peripapillary halo. Total obscuration on the disc of a segment of a major blood vessel.

Stage 5 - Severe papilledema dome-shaped protrusions representing an anterior expansion of the optic nerve head. The peripapillary halo is narrow and smoothly demarcated. Total obscuration of a segment of a major blood vessel may or may not be present. Obliteration of the optic cup.
